# Supplementary material for: Predictive factors for readmission due to neonatal hyperbilirubinemia: A retrospective case-control study
Source: PLoS One. 2025 Apr 1;20(4):e0320767. doi: 10.1371/journal.pone.0320767 (PMC11960944; doi:10.1371/journal.pone.0320767)
Supplement: Attachment — Submitted filename: Response to Reviewers.docx [file pone.0320767.s003.docx]

**Dear Editor and Reviewers,**

Thank you very much for taking the time to review our manuscript, titled "[Analysis of Readmission Risk Factors for Neonatal Hyperbilirubinemia] (PONE-D-24-45603)." We are sincerely grateful for your constructive comments and valuable suggestions, which have been instrumental in improving the quality of our work.

In response to your feedback, we have carefully revised the manuscript and provided detailed responses to each of the reviewers' comments below. We truly appreciate your efforts and thoughtful recommendations, and we hope the revisions meet your expectations.

Thank you once again for your time and consideration.

Abstract section:

- Write abbreviations in full in their first use in the text. Eg. Total Serum Bilirubin (TSB)

**Response**：Thank you so much for your feedback! I truly appreciate you pointing this out. I will make sure to write all abbreviations in full when they are first mentioned, such as Glucose-6-Phosphate Dehydrogenase（G6PD）and Total Serum Bilirubin (TSB). Your input is very valuable, and I’ll keep this in mind moving forward!

Introduction section:

- Which is the appropriate term, “nuclear jaundice Vs Kernicterus”.  Page- 1, paragraph -1, line -4.

**Response**：Thank you so much for pointing this out! The term kernicterus is more commonly used in medical literature and practice to describe this condition. we have decided to use the term 'Kernicterus' and have updated the text accordingly. We truly appreciate your thoughtful feedback and will continue to strive for clarity and accuracy in our work!

- I appreciate the conciseness of the introduction section. However, it will be good if the authors mention findings of previous similar studies.

**Response**：Thank you very much for your valuable feedback! We greatly appreciate your positive comments about the clarity of the introduction. We agree that referencing findings from previous similar studies would strengthen the context and background of our study. In the revised manuscript, we will incorporate relevant literature to highlight the findings of similar studies and provide a more comprehensive foundation for our research. Thank you again for your constructive suggestion, which will help improve the overall quality of the manuscript!

Participants and methods section:

- Why gestational age of 35 weeks is taken as cut-off point for inclusion in the study? Why not 34 weeks or 37 weeks?

**Response**：Thank you very much for your thoughtful question! We chose a gestational age of 35 weeks as the cut-off point for inclusion in the study based on recommendations from the American Academy of Pediatrics (AAP) and other international guidelines. These guidelines typically apply treatment standards, such as bilirubin thresholds for phototherapy and exchange transfusion, to infants born at ≥35 weeks, making this cut-off clinically relevant and aligned with international standards.

Infants born at 35 weeks and above fall into the category of 'late preterm infants,' who have significant differences in bilirubin metabolism compared to full-term infants (≥37 weeks). Clinically, this group is a key focus for hyperbilirubinemia management. Our study aims to identify predictive factors for readmission due to neonatal hyperbilirubinemia. By selecting 35 weeks as the lower limit, we can better focus on a population with relatively immature bilirubin metabolism while still having a certain level of developmental maturity (late preterm and near-term infants).

This threshold ensures that our research objectives align closely with real-world clinical concerns. Additionally, several studies, such as those by Xu et al. (2023) and Belide et al. (2023), have also used ≥35 weeks as the inclusion criteria for studying hyperbilirubinemia-related issues. Aligning with these studies not only enhances the comparability of our results but also increases their clinical relevance and applicability.

We truly appreciate your insightful question, and we hope this explanation clarifies the rationale behind our decision. Your feedback helps us ensure our methodology is transparent and well-justified!

- Babies born at 35 weeks do not have the same bilirubin metabolism capacity to that of babies born at term. Have you controlled for the variable “gestational age” in the analysis when you are comparing for the effect of other variables between the two groups?

**Response**：Thank you very much for your insightful question! We fully agree that gestational age plays a crucial role in bilirubin metabolism, and we have carefully considered this in our analysis.

In our multivariable logistic regression model, we included gestational age as an independent variable to assess its impact on the risk of readmission for neonatal hyperbilirubinemia, while controlling for its potential confounding effects on other variables, such as ABO hemolysis and G6PD deficiency. In the initial draft of the model, gestational age was explored both as a continuous variable and as a categorical variable. However, to ensure the accuracy of the regression model and to avoid redundancy from using the same variable in different forms, we revised the model and retained gestational age as a categorical variable (preterm <37 weeks vs. term ≥37 weeks). This allowed us to specifically analyze whether preterm birth—a clinically recognized high-risk group—is an independent risk factor for readmission.

We also conducted multicollinearity tests for gestational age and related variables, such as birth weight, using tolerance and the variance inflation factor (VIF). The results confirmed that there was no significant multicollinearity between gestational age and other variables.

In the adjusted model, preterm birth (<37 weeks) was identified as an independent risk factor for readmission (OR=9.541, p＜0.001). Importantly, even after controlling for gestational age, other variables such as ABO hemolysis, G6PD deficiency, and high TSB levels remained significant independent predictors. This indicates that the effect of gestational age has been effectively accounted for, and the results for the other variables are robust.

We sincerely appreciate your thoughtful feedback, which helped us ensure that our analysis is both thorough and clear. We hope this explanation addresses your concern, and we are happy to provide further details if needed!

- The authors used the term “Retrospective” to describe their study design. However, the term “retrospective” doesn’t describe type of study design and it only tells us that the data used in the study is past time event. Therefore, it is better to use terms such as “retrospective cross-sectional study” or “retrospective cohort study” or “case-control study” design as deemed necessary.

**Response**：Thank you very much for your valuable suggestion! Your explanation regarding the term 'retrospective' is absolutely correct—we fully understand that it only indicates that the data were collected from past events and does not specifically describe the study design.

In our study, we actually adopted a **retrospective case-control study** design. We retrospectively collected past case data and categorized participants into a case group and a control group based on whether they were readmitted due to hyperbilirubinemia. Subsequently, we analyzed the associated risk factors. In our revised manuscript, we will ensure that the term 'retrospective case-control study' is used to more accurately reflect the specific study design and avoid potential ambiguity.

Once again, thank you for your constructive feedback. We have already begun making the necessary revisions and are more than happy to consider any additional suggestions you may have to further improve our study!

- Provide explanation why you excluded babies with congenital malformation, hypoxia, infectious disease…. from the study while you included babies with G6PD deficiency and ABO hemolytic disease.

**Response**：Thank you for your thoughtful question! We greatly appreciate your interest and would like to provide a detailed explanation of our reasoning.

In this study, we excluded infants with congenital anomalies, hypoxia, and infectious diseases because these factors can have broad and uncontrollable effects on the risk of neonatal hyperbilirubinemia and subsequent readmission. For instance,congenital anomalies may be associated with other metabolic disorders, hypoxia can impair bilirubin metabolism, and infectious diseases can significantly alter the mechanisms of bilirubin production and clearance. Including these infants could confound the analysis of risk factors related to hyperbilirubinemia, potentially reducing the reliability and interpretability of the results.

On the other hand, we included infants with G6PD deficiency and ABO hemolytic disease because these are well-established and direct etiological factors associated with neonatal hyperbilirubinemia. One of the objectives of our study was to evaluate whether these specific factors independently contribute to the risk of readmission for hyperbilirubinemia. Including these infants allows us to provide more robust evidence on the role of these conditions in the context of neonatal hyperbilirubinemia.

We sincerely thank you for this insightful question, as it gives us an opportunity to clarify the rationale behind our study design. If you have any further questions, we would be happy to discuss them in more detail!

- In the introduction section, the authors have stated that the aim of the study was to determine “…risk factors for readmission…”. However, it appears that the authors implemented a “Retrospective cross-sectional” study design which is not suitable to determine temporal relationship between variables which is important to determine “risk factors”. Cross-sectional study allows to determine “association” between two variables and doesn’t allow determination of “risk factors”. Moreover, “risk” is calculated by using “incidence rate” which not assessed in the current study. Therefore, I recommend the authors to think about the appropriateness of the use of the term “…risk factor…”. Also, think about the title; which one is appropriate title for the data set you have used: “analysis of readmission risk factors” Vs “Predictors of readmission”?

**Response**：Thank you very much for your valuable feedback! Your analysis of the use of the term 'risk factors' and the limitations of the study design is highly insightful, and we deeply appreciate your input.

This study utilized a **retrospective case-control design**, which compares cases (readmitted infants) and controls (non-readmitted infants) to identify factors associated with readmission. While case-control studies are effective for identifying associations between variables and outcomes, they are indeed limited in establishing temporal relationships between variables, which is a critical requirement for defining 'risk factors.'

Based on your suggestion, we agree that the term 'predictive factors' is more appropriate for describing our findings, as our analysis primarily aims to identify factors that can predict the risk of readmission for neonatal hyperbilirubinemia. We will adjust both the article title and the terminology used throughout the manuscript to ensure they more accurately reflect the study design and objectives, thereby avoiding any potential misunderstanding.

Once again, thank you for your thoughtful and constructive feedback. It has provided us with clear directions for improvement. If you have any further suggestions, we would be more than happy to discuss and incorporate them!

- The authors mentioned that “Phototherapy and exchange transfusion are performed in accordance with established guidelines.” I advise the authors to cite these guidelines so that readers can refer them.

**Response**：Thank you very much for your valuable suggestion! We completely agree that citing the relevant guidelines would provide readers with more comprehensive reference information. In this study, phototherapy and exchange transfusion were performed in accordance with the **American Academy of Pediatrics (AAP) Guidelines for Management of Hyperbilirubinemia** (2004 version). We will ensure to explicitly reference these guidelines in the revised manuscript and include detailed citations for readers to consult.

Once again, thank you for your constructive feedback, which will help improve the transparency and rigor of our study. If you have any further suggestions, we are more than happy to incorporate them!

- Move the “ethical approval statement” to the end of the methods section.

**Response**：Thank you for your suggestion! We understand the importance of placing the ethical approval statement in the most appropriate section for better readability and alignment with standard manuscript structures. We will move the ‘ethical approval statement’ to the end of the methods section in the revised manuscript as per your recommendation. We truly appreciate your feedback to improve the organization of the paper!

Table 1:

- Abbreviations used the table should be written in full or should be written in expanded form as foot note
- The variable “Resuscitation at Birth” has “zero value” in all the cells in the table. Therefore, there is no need to list this variable in the table.

**Response**：Thank you for your valuable suggestions!

1. We agree that abbreviations used in the table should either be written in full or expanded as footnotes for clarity. We will revise the table accordingly in the updated manuscript.
2. Regarding the variable 'Resuscitation at Birth,' we appreciate your observation. Since all cells have a 'zero value,' we agree that this variable does not add relevant information to the table. We will remove it from the table in the revised version.

We truly appreciate your constructive feedback, which helps improve the clarity and presentation of the manuscript!

Table 2:

- The variables “Exchange transfusion” and “Rh hemolysis” have “zero value” in all the cells in the table. Therefore, there is no need to list these variables in the table.

**Response**：Thank you very much for your valuable observation!

We agree that the variables 'Exchange transfusion' and 'Rh hemolysis' have zero values in all the cells, and their inclusion does not contribute additional information to the table. We will remove these variables from the table in the revised version of the manuscript to improve its clarity and relevance.

We truly appreciate your constructive feedback and will ensure the table is updated accordingly. Thank you again for helping us enhance the quality of our manuscript!

Table 3:

- Table-3 lacks clarity since it is not clear how some of the variables are categorized. For example, how is the variable “Birth weight” is categorized and its Odds Ratio is calculated?

**Response**：Thank you for raising this important question about the treatment of the 'birth weight' variable in our analysis.

In the initial draft, 'birth weight' was included in the regression model as a continuous variable, analyzed with each 1-gram increment as the unit of change. The odds ratio (OR) reflected the impact of 'birth weight per 1-gram decrease' on the risk of readmission, with an OR close to 1 (OR=0.998, p=0.054), suggesting a relatively small effect of birth weight in the analysis.

To improve the clinical interpretability of the results, we also attempted to categorize birth weight into three groups:

- **Low birth weight group (<2500g):** A high-risk group associated with prematurity and low weight.
- **Normal weight group (2500–4000g):** The typical weight range for term infants.
- **High birth weight group (>4000g):** A group potentially associated with an increased bilirubin metabolism burden.

However, upon reviewing the distribution, we found that the low birth weight group and high birth weight group each only contained 4 cases, while the vast majority of cases fell into the normal weight group. Due to the extremely small sample sizes in the low and high birth weight groups, including this categorization in the regression model would likely result in:

1. **Unstable statistical results:** The ORs and confidence intervals for the small groups would likely be very unstable and potentially extreme, reducing the reliability of the results.
2. **Limited statistical and clinical significance:** The uneven sample distribution would reduce statistical power, and results derived from such small groups would have limited generalizability in clinical practice.
3. **Reduced model credibility:** The inclusion of small groups increases the risk of overfitting, which could obscure meaningful results from larger groups.

Given these considerations, we opted to retain birth weight as a continuous variable in the regression model, as this approach is more robust and suitable for the dataset. In the revised manuscript, we will provide a detailed explanation of the categorization attempt and the rationale for ultimately treating birth weight as a continuous variable. This will ensure transparency and help readers clearly understand the logic behind our analytical choices.

Thank you again for your insightful feedback, and we are happy to address any further questions you may have!

- The variable “Gestational age” is included in the regression model twice (as continuous variable and as a categorical variable). Such inclusion of a single variable in different form distorts the regression model and the authors either have to remove one form of the variable or provide plausible explanation for doing so.

**Response**：Thank you very much for your valuable feedback! We completely understand that including the same variable in both continuous and categorical forms in the regression model could potentially affect the model's accuracy. We have carefully considered this issue.

In the initial draft, we included 'gestational age' in both continuous and categorical forms in the model to explore the impact of this important variable on the risk of readmission from different perspectives. Upon comparison, we found some overlap in the results between the two forms. However, the continuous form was better suited for capturing subtle changes in gestational age's effect on readmission risk, while the categorical form (e.g., preterm <37 weeks vs. term ≥37 weeks) provided greater clinical interpretability.

Recognizing that using the same variable in different forms could compromise the stability and accuracy of the model, we have revised the analysis in the updated manuscript. We chose to retain the categorical form of 'gestational age' (preterm vs. term) and removed the continuous form. This decision was based on the following considerations:

1. **Stronger clinical interpretability**: Clinicians in actual practice are more concerned with preterm infants as an independent high-risk group rather than the subtle effects of weekly changes in gestational age on risk. Using the categorical variable (preterm vs. term) aligns better with this clinical perspective.
2. **Avoiding multicollinearity**: Including both continuous and categorical forms of the same variable could lead to multicollinearity, potentially affecting the accuracy of the results.

After removing the duplicate variable, we will re-evaluate multicollinearity for the remaining independent variables (e.g., birth weight, G6PD deficiency) to ensure the final model's robustness and accuracy.

We will clearly explain this decision and rationale in the revised manuscript. Once again, thank you for your constructive feedback on improving the accuracy of the model. If you have further suggestions, we are more than happy to discuss and refine the analysis further!

- The variables “Gestational age” and “birth weight” appears to have collinearity. However, the authors included both variables in the model while collinear variables shouldn’t be included without checking that they are no collinear.

**Response**：Thank you for your valuable feedback! We highly value the potential impact of collinearity on the stability and accuracy of the regression model. As such, we conducted collinearity tests for the variables 'gestational age' and 'birth weight' during the model construction process.

In the initial draft, both 'gestational age' and 'birth weight' were included in the model. Additionally, gestational age was included in both continuous and categorical forms. To ensure the accuracy of the regression model and avoid duplicate use of the same variable in different formats, we revised the model to retain only the categorical form of gestational age (preterm <37 weeks vs. term ≥37 weeks). This approach allowed us to specifically analyze preterm infants as a clinically recognized high-risk group and evaluate whether they represent an independent risk factor for readmission.

After adjusting the model, we re-evaluated the potential collinearity between gestational age and related variables (such as birth weight) using tolerance and the variance inflation factor (VIF). The results indicated that there was no significant collinearity between gestational age and other variables, confirming that it is appropriate to include both variables in the model.

Additionally, the decision to include both variables is based on their distinct clinical significance:

1. **Gestational age**: Primarily reflects the maturity of the newborn, which has a direct impact on bilirubin metabolism and the risk of readmission.
2. **Birth weight**: Mainly reflects the developmental level of the newborn's physical growth and is associated with the bilirubin metabolism burden and the risk of readmission.

In the revised manuscript, we will provide a more detailed explanation of the collinearity testing process and results to enhance the transparency of the methods section.

Thank you again for your insightful feedback! If you have further suggestions, we are more than happy to continue refining our analysis and improving the manuscript.

- What is the basis of comparing the variables “preterm birth <37 wks” vs “Term birth”? We know that preterm babies are at increased risk of hyperbilirubinemia due to immaturity of UDGT enzyme.

**Response**：Thank you very much for your insightful question! We completely agree that preterm infants are more prone to developing hyperbilirubinemia, which is closely related to the immaturity of UDP-glucuronosyltransferase (UDPGT) enzyme. In this study, the decision to compare variables based on 'preterm <37 weeks' and 'term-born infants' was made on the following clinical and research grounds:

1. **Clinical significance**: Preterm birth (<37 weeks) is a widely recognized high-risk classification in clinical practice. Preterm infants, due to the immaturity of the UDPGT enzyme, have reduced bilirubin metabolism capacity. Additionally, they often exhibit other physiological and metabolic immaturities, such as decreased bilirubin clearance rates and underdeveloped liver function, making them a high-risk group that requires focused attention.
2. **Guideline recommendations**: The American Academy of Pediatrics (AAP) and other international guidelines distinguish infants born at 35–37 weeks (late preterm) from term infants, using this classification as the basis for different management strategies and treatment thresholds. This highlights the independent clinical significance of infants born at <37 weeks in the context of jaundice management.
3. **Study design and data distribution**: An analysis of our study data showed that using 'preterm <37 weeks' as the cutoff effectively differentiates the high-risk group (preterm infants) from the lower-risk group (term infants), allowing for a more precise analysis of the independent impact of prematurity on the risk of readmission.
4. **Comparability and reference value**: Defining preterm as <37 weeks aligns with the standard used in most international studies. This ensures that our findings are comparable with global research and adds reference value to related studies worldwide.

In the revised manuscript, we will elaborate on the rationale for selecting 'preterm <37 weeks' and 'term-born infants' as the basis for variable comparison in the methods section. Once again, thank you for your valuable feedback on our study design! If you have further suggestions, we would be happy to discuss and improve our work further.
